# Dynamic Detection of Specific Membrane Capacitance and Cytoplasmic Resistance of Neutrophils After Ischemic Stroke

**DOI:** 10.14336/AD.2023.0127

**Published:** 2023-08-01

**Authors:** Haiping Zhao, Xiaofeng Luan, Yuqing Wang, Yifei Ye, Feng Yan, Xue Li, Yuang Li, Mingxiao Li, Lingqian Zhang, Yang Zhao, Chengjun Huang, Yumin Luo

**Affiliations:** ^1^Institute of Cerebrovascular Diseases Research, Xuanwu Hospital of Capital Medical University, Beijing, China.; ^2^Beijing Geriatric Medical Research Center, Beijing, China.; ^3^Institute of Microelectronics, Chinese Academy of Sciences, Beijing, China.; ^4^University of Chinese Academy of Sciences, Beijing, China.

**Keywords:** stroke, microfluidic impedance flow cytometry, instantaneously characterization, cellular electrical properties, specific cell membrane capacitance

## Abstract

Peripheral blood is the most readily available resource for stroke patient prognosis, but there is a lack of methods to detect dynamic changes of neutrophils in peripheral blood that can be used in the clinic. Herein, we developed a procedure to characterize dynamic changes of neutrophils based on their electrical properties in rats after transient middle cerebral artery occlusion (MCAO). We characterized the specific membrane capacitance (C_sm_) and cytoplasmic resistance (σ_cyto_) of approximately 27,600 neutrophils from MCAO rats 24 h after ischemia/reperfusion. We found that the C_sm_ and σ_cyto_ of neutrophils in the MCAO group were significantly higher compared to the sham group. Furthermore, we observed a monotonically upward shift in neutrophil Csm in the MCAO group during the four 5-minute test cycles. Our findings suggest that the dynamic changes of cellular electrical properties could reflect neutrophil activity and serve as a prognostic indicator for ischemic stroke in the clinical setting.

Acute ischemic stroke has a poor prognosis with limited predictors and therapeutic options [1]. Peripheral blood is the most readily available resource for clinical prognosis [2]. The neutrophil ratio has been preliminarily used as a prognostic indicator for ischemic stroke in the clinical setting [3]. However, conventional blood cell analysis methods cannot detect the dynamics of neutrophils in peripheral blood. Furthermore, neutrophil activity plays a crucial role in the prognosis of ischemic stroke [3], but current rapid analytical methods to assess neutrophil activity at multiple timepoints following stroke are lacking.

At the single-cell level, flow cytometry has conventionally been used in experimental research to determine neutrophil activity [4]. However, this method has limited clinical application for the prognosis of acute ischemic stroke due to the time-consuming labeling procedure. Recently, single cells' inherent electrical properties [5] (e.g., specific membrane capacitance (C_sm_) and cytoplasmic resistance (σ_cyto_)) have been used to classify peripheral blood immune cells [6] and monitor changes in the tumor cell status [7] in a label-free manner.

Such mothed could furtherbe used to explore whether the electrical properties of living neutrophils from blood could detect any differences between stroke and sham groups. Considering that the neutrophils are undergoing a dynamic process and their label-free inherent electrical properties could be rapidly measured, we developed a new paradigm of multi-timepoint characterizations and trend analysis of the neutrophil based on neutrophil electrical properties, which could be used as a prognostic indicator for paitentis with ischemic stroke.

## Characterization of C_sm_ and σ_cyto_ of neutrophils from cerebral ischemia-reperfusion and sham-operated rats

For these experiments, Sprague-Dawley rats subjected to middle cerebral artery occlusion (MCAO) were used as an ischemic stroke model (Supplementary data). After two hours of cerebral ischemia and 24 hours of reperfusion, neutrophils were collected and separated from the peripheral blood of the rats using gradient density centrifugation (Supplementary data). The separated neutrophils from MCAO and sham-operated rats were further utilized to characterize C_sm_ and σ_cyto_ using a high-throughput single-cell electrical property characterization method [7]. Single cells were driven to pass continuously through the cross-shaped constriction microchannel of the microfluidic impedance flow cytometer accompanied by impedance monitoring. The raw impedance changes were translated into C_sm_ and σ_cyto_ values using an equivalent circuit model (Please see Supplementary data for the detail).

**Figure 1.**
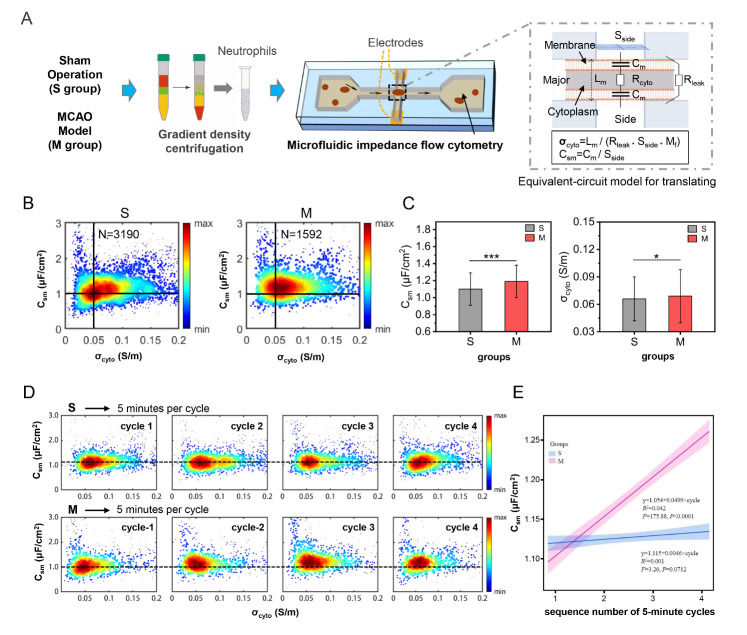
**Electrical properties of neutrophils from the rat model of middle cerebral artery occlusion (MCAO) and sham rat**. (**A**) Flow chart for detecting specific membrane capacitance (C_sm_) and cytoplasmic resistance (σ_cyto_) of the neutrophils derived from the MCAO and sham rats. (**B**) Scatter plots and (C) Histograms of C_sm_ and σ_cyto_ of neutrophils from the MCAO and sham groups at the third cycle. (**D**) Scatter plots and (E) Different rising trends of C_sm_ and σ_cyto_ of four 5-minute cycles of the neutrophils from the MCAO and sham groups.

## Electrical properties of neutrophils isolated from the MCAO and the sham groups at single timepoints

Figure 1A shows a flow chart to characterize the electrical properties of neutrophils from MCAO and sham rats. Figure. 1B shows the strical properties of the neutrophils isolated from MCAO group and the sham group at a single timepoint, represented by cycle 3. Slight differences were observed in both C_sm_ and σ_cyto_ of the neutrophils from the MCAO and sham groups (Fig. 1C).

## The increasing trend of the electrical properties of neutrophils after MCAO

We characterized the C_sm_ and σ_cyto_ of approximately 27,600 neutrophils from MCAO rats 24 h after ischemia/reperfusion and sham groups (n=6 per group). To explore the different variation trends of the electrical properties of neutrophils from the MCAO and sham groups, we performed four continuous 5-minute characterization cycles for each rat. Surprisingly, with the prolongation of the isolation time, the C_sm_ in the MCAO group showed a gradual upward shift tendency in the scatter plots of four measurement cycles, but relatively stable in the sham group (Fig. 1D). After linear regression analysis, a significant rising trend was found in the MCAO group with a slope of 0.0499μF/cm^2^/cycle (*P*<0.0001), compared with the sham group (k=0.0046μF/cm^2^/cycle, *P*=0.0712) (Fig. 1E).

Collecitvely, we reported that the rising trend of four cycles was more visible compared with the absolute value obtained at single timepoints. Considering the trend could be observed by self-referencing in practice, it was more robust to be exploited than the single timepoint sampling biomarkers. Furthermore, previous findings regarding the upregulation of membrane capacitance (C_m_) are worth extended. Firstly, the mobilization of subcellular granules to the cell membrane is followed by a change in C_m_ in neutrophils. An increase in C_m_ induced by GTPγS in single human neutrophils has been studied using a patch-clamp during exocytosis dynamics [8]. Secondly, granule-specific ATP requirements for Ca^2+^-induced exocytosis of granule subsets in human neutrophils have been shown to depend on changes in C_m_ [9]. Then, the observed increase in C_sm_ may reflect the process of neutrophil degranulation, which is a critical pathological process to influence the clinical outcomes of patients with ischemic stroke. Therefore, single-cell bioelectrical phenotyping may be associated with early clinical prediction of stroke outcomes. In conclusion, these findings provide a new perspective to investigate the molecular mechanism of neutrophil degranulation and could provide a platform for screening potential pharmacological therapies.

## Supplementary Materials

The Supplementary data can be found online at: www.aginganddisease.org/EN/10.14336/AD.2023.0127.
