# Supplementary material for: Dynamic Detection of Specific Membrane Capacitance and Cytoplasmic Resistance of Neutrophils After Ischemic Stroke
Source: Aging Dis. 2023 Aug 1;14(4):1035–7. doi: 10.14336/AD.2023.0127 (PMC10389826; doi:10.14336/AD.2023.0127)
Supplement: Supplementary file 1 — The Supplementary data can be found online at: www.aginganddisease.org/EN/10.14336/AD.2023.0127. [file AD-14-4-1035-s.pdf]

SUPPLEMENTARY DATA

Dynamic Detection of Specific Membrane Capacitance and Cytoplasmic Resistance of Neutrophils After Ischemic Stroke

Haiping Zhao, Xiaofeng Luan, Yuqing Wang, Yifei Ye, Feng Yan, Xue Li, Yuang Li, Mingxiao Li, Lingqian Zhang[3], Yang Zhao, Chengjun Huang, Yumin Luo

© 2023. Zhao H et al. Published online at https://www.aginganddisease.org/EN/10.14336/AD.2023.0127

SUPPLEMENTARY DATA

MATERIALS AND METHODS

Establishment of a rat cerebral ischemia-reperfusion model

Sprague-Dawley rats (male, 280–300 g) were obtained from Vital River Laboratory Animal Technology Co., Ltd. (Beijing, China). All animal studies were approved by the Institutional Animal Care and Use Committee of Capital Medical University (XW-20211130-1, Beijing, China) and were carried out according to international and national laws and policies. Rats subjected to middle cerebral artery occlusion (MCAO) were used as an experimental stroke model (n=3). Anesthesia was induced with enflurane mixed with 70% N_2O and 30% O_2 and maintained with 2% enflurane mixed with 70% N_2O and 30% O_2. A midline incision was made in the neck to separate the right common carotid artery from the internal and external carotid arteries. A nylon suture (diameter, 0.38 mm) was used to insert the internal carotid artery from the stump of the external carotid artery to the distance between the internal carotid artery and the external carotid artery. The artery bifurcation was approximately 2 cm, and the ischemia time was 2 h. The rectal temperature of the rats was monitored using a feedback temperature control blanket and the body temperature of the rats was maintained between 36.5℃ and 37.5℃. The nylon fishing line was not inserted into the sham-operated control group (n=3) and the rest of the steps were the same as those in the operation group. After 2 h of ischemia/24 h of reperfusion, the abdominal main venous blood was collected, and neutrophils were isolated for characterization of electrical properties. Brain tissues were subjected to TTC staining to identify cerebral infarction.

Separation of peripheral blood neutrophils from MCAO and sham rats

Neutrophils were obtained from the blood of rats in the cerebral ischemia and sham groups by the dextran precipitation method combined with the Ficoll density gradient centrifugation method following the instructions in the manual (Tianjin Haoyang Biological Products Technology Co., Ltd. Responsible Company, 2010X1118). The neutrophils were mixed with 300 µl phosphate buffer and filtered using a 40 µm sterile filter. The cell concentration was adjusted to 6×10^5 cells/ml, and the cells were used for the subsequent characterization of the electrical properties.

Setup of Impedance flow cytometry and microfluidic device fabrication

Impedance flow cytometry was performed as previously described [1]. In summary, it was composed of a microfluidic chip with a crossing constriction channel, a lock-in amplifier (model 7270, Signal Recovery, USA) to record impedance changes, a function generator (WF 1948, NF, Japan) as the signal source, a pneumatic pressure controller (Pace 5000, Druck, USA), a data acquisition card to translate the analogical signal to digital wave data for the data processing program, and an inverted biological fluorescence microscope (Olympus IX73, Olympus, Japan).

A cross-shaped constriction microchannel was designed as the core sensing unit for impedance flow cytometry. The cross-sectional area of the cross-shaped constriction microchannels was designed to be smaller than that of the target cells. During characterization, cells were aspirated to continuously pass through the major constriction channel with a negative pressure applied to the output of the main channel. When the cells reached the point of intersection, the narrow microchannel design tightly blocked the cells in the mouths of both unilateral constriction channels to form a high resistance sealing to improve sensitivity. The crossing constriction channel included a major channel (6 µm in width and 6 µm in height) for cell passage and a side channel (3 µm in width and 6 µm in height) to connect to a pair of planar electrodes located on the substrate at both ends of the side channel. The manufacturing method of the microfluidic chip was previously introduced [1]. The microfluidic chip was manufactured by combining a PDMS (SYLGAR 184, Dow, USA) cover with a crossing constriction channel to a glass substrate with the planar electrodes patterned on its top surface. The PDMS cover was duplicated from a male mold mask that featured a two-layer channel structure. The first layer of the crossing constriction channel was fabricated using a deep reactive ion etching microfabrication process to form a 6 µm high microchannel, and the SU-8 photoresist was patterned through exposure and development to form the second layer of the cell loading channel with a height of 30 µm. The planar gold electrodes on the glass substrate were patterned using a lift-off microfabrication process. As the manufacturing procedure of the microfluidic chip is shown in Figure S1, the Au metal was sputtered on the photoresist-patterned substrate. Then, the Au covered on the photoresist was removed with the photoresist, leaving the patterned Au lines on the glass substrate as planar electrodes. After treatment with oxygen plasma for approximately 3 min, the PDMS cover was bonded to the substrate to form the microfluidic chip.

© 2023. Zhao H et al. Published online at https://www.aginganddisease.org/EN/10.14336/AD.2023.0127

SUPPLEMENTARY DATA

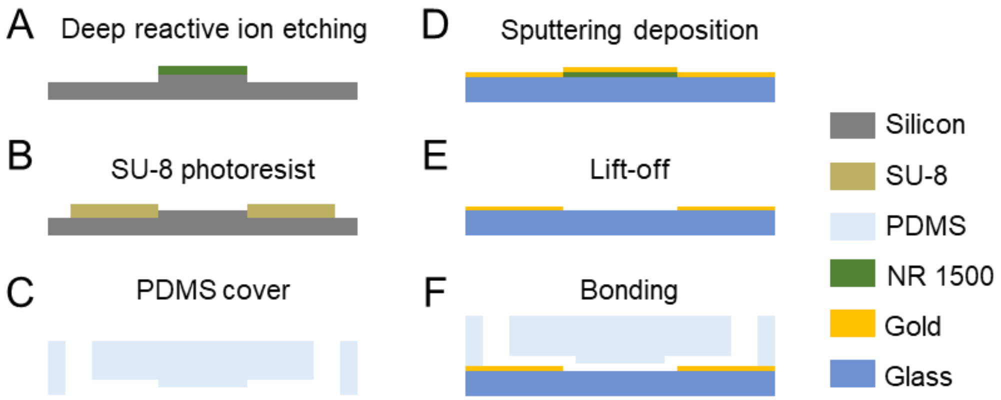

Supplementary Figure 1. The manufacturing workflow of the microfluidic chip. The male mold mask is manufactured using a deep reactive ion etching microfabrication process (A) and SU-8 photoresist (B). The PDMS cover is duplicated from the mask (C). The lift-off microfabrication process (D and E) is used to manufacture the gold planar electrodes on the glass substrate. Bonding the PDMS cover to the glass substrate (F).

Electrical properties characterization of neutrophils

Electrical properties of peripheral blood neutrophils from six rats in the cerebral ischemia model group and the sham-operated control group were characterized. Similar to the procedure reported in our previous study, a negative value of 3 kPa was applied in the characterization process to continuously drive single cells through the major constriction channel. A dual-frequency (0.5 V, 100, and 180 kHz) AC sinusoidal signal was applied to the microfluidic chip as an excitation signal. The impedance of the channel without the cell $Z_{channel}$ was composed of the parasitic capacitance C_p and the resistance of the medium R_{ch}. When a single cell passes through the intersection of the channel, the total impedance of the channel Z_{total} changes due to the relative insularity of the cell, which can be expressed as a couple of the $Z_{channel}$, Z_{cell}, and the leakage resistance R_{leak} (reflects the current path around the cell). The impedance monitoring unit recorded the impedance changes between the paired electrodes. Combined with the equivalent electrical model proposed previously [1, 2], the intrinsic electrical characteristics of the cell (C_{sm} and σ_{cyto}) can be obtained. The duration of each test cycle was set at 5 min, and several hundred to 1000 cells were recorded.

Statistics

Using GraphPad Prism Vision 8.0.1 (GraphPad Software, San Diego, CA, USA), the differences of thousands of neutrophils between the Sham and MCAO groups were compared using an unpaired two-tailed Student's t-test. Data are represented as mean ± SD. With JMP Pro 16.0.0 (SAS Institute Inc., Cary, NC, USA), simple linear regression was used to model the relationship between cycle and C_{sm} in the Sham and MCAO groups. A *P*-value of < 0.05 was considered statistically significant.

References

[1] Zhao Y, Wang K, Chen D, Fan B, Xu Y, Ye Y*, et al.* (2018). Development of microfluidic impedance cytometry enabling the quantification of specific membrane capacitance and cytoplasm conductivity from 100,000 single cells. Biosens Bioelectron, 111:138-143.

[2] Luan XF, Li Y, Zhao HP, Sun S, Fan YY, Zhang WC*, et al.* (2022). Discovery of the correlation between the suspended membrane capacitance and adherent morphology of single cells enriching from clinical pleural effusion revealed by a microfluidic impedance flow cytometry. Sensors and Actuators B-Chemical, 371.

© 2023. Zhao H et al. Published online at https://www.aginganddisease.org/EN/10.14336/AD.2023.0127
